# Evaluating the appropriateness of γ-graphyne derivatives as electrode materials for supercapacitors

**DOI:** 10.1038/s41598-023-41637-w

**Published:** 2023-09-12

**Authors:** Mahsa Abbasi Kenarsari, Mohsen Vafaee, Mokhtar Nasrollahpour, Seyyed Morteza Mousavi Khoshdel

**Affiliations:** 1 Department of Chemistry, Tarbiat Modares University (TMU), P.O. Box 14115-175, Tehran, Iran; 2 Department of Chemistry, Iran University of Science and Technology, P.O. Box, Tehran, 16846-13114 Iran

**Keywords:** Physical chemistry, Theoretical chemistry, Computational chemistry, Density functional theory, Energy

## Abstract

DFT calculations were used to study the quantum capacitance of pure, B/Al/Si/N/P-doped, and defective γ-graphyne. Due to the direct relationship between capacitance and electronic states around the Fermi level, structures' electronic properties were evaluated by DOS plots. The results of integrated specific quantum capacitance in the range of water stability potential show an improvement of capacity in each p and n-type doping. The calculated cohesive energies of doped structures reflect the stability enhancement. Also, the stability/capacitance of single and double vacancies in two distinct positions (sp and sp2) were examined. The results illustrate stability retention and quantum capacitance improvement of these defective structures. Among the doped structures, the maximum quantum capacitance is 2251.10 F/gr belonging to the aluminum doped structure (in the sp position). For the defective structures, the maximum quantum capacitance is 4221.69 F/gr belonging to removing two sp carbon atoms. These quantum capacitances significantly improved compared to the pristine structure (1216.87 F/gr) and many other structures. These stunning results can contribute to the design of appropriate structures as electrode materials for high-efficiency supercapacitors.

## Introduction

Due to the changing global landscape, fossil fuel depletion and expanding costs, air contamination, global warming, and increasing demand for portable systems and hybrid electric vehicles, researchers are encouraged to design more efficient energy storage devices^1,2^. Thus, developing electrochemical energy conversion and storage devices with high performance, low cost, and environmentally friendly for powering an increasingly diverse range of applications is one of the most critical challenges of today's dynamic society^3–6^.

Batteries, fuel cells, and supercapacitors are effective and practical electrochemical energy conversion and storage technologies^7,8^. Among these, supercapacitors are significant because of their high power density, not having memory effect, long life cycle, fast charging/discharging rate, good stability, and wide operating temperature range^9–11^.

Recently, carbon-based materials, such as activated carbons (AC)^12^, carbon nanotubes (CNTs)^13^, porous carbons (PCs)^14,15^, graphene, and its derivatives^16,17^, for example, graphyne (GY)^18^ and graphdiyne (GDY)^19^ have been investigated and utilized as electrode materials for supercapacitors. These non-poisonous materials have a high specific surface area, high electrical conductivity, a simple creation process, and proper resistance^20^. They have an adjustable energy gap and can maintain stability over wide temperature ranges. These features make them appropriate for use as the electrode of supercapacitors, but they suffer from low quantum capacitance with all these advantages^21,22^. In addition, they have a limited specific capacitance that outcomes from small quantum capacitance and, in fact, a lack of states near the Fermi level.

With some chemical modifications, Stoller et al.^23^ exploited graphite to graphene oxide (GO) for use as electrodes for a supercapacitor and expressed that their chemically modified graphene has good electrical conductivity, high surface area, and could be a hopeful candidate for electrochemical double layer capacitors (EDLCs)^24^. Also, Graphene papers have attracted much attention for electrodes of flexible supercapacitors due to their adjustable thickness, flexibility, and required electrical properties^25,26^. Mousavi et al. achieved significant quantum capacitances by doping and co-doping some adatoms on graphene and functionalization it for use as an electrode in asymmetric and symmetric supercapacitors^4,27^.

Graphyne is a two-dimensional (2D) structure proposed theoretically in 1987 by Bagman et al.^28^ with one atom thickness and a framework of sp and sp^2^ carbon atoms. In 2018, this structure was synthesized by Qiaodan et al.^29^. Their results confirmed the theoretical calculations by Bagman et al., such as semiconductor character and lattice parameter of 0.69 nm. The different allotropes of graphyne can be acquired by embedding acetylenic linkages into pure graphene^30^. As per this, α-, β-, δ, and γ-graphyne contain 100%, 66.67%, 41.67%, and 33.33%, of acetylenic groups in their structure, respectively. Acetylenic linkages make graphyne a material with fascinating structural, mechanical, thermal, optical, and particularly electrical properties. For instance, compared to graphene, the pore size of graphynes increases because of the acetylene bond, which decreases the average coordination number. Along these lines, graphyne pore size is more suitable for ion adsorption in electrolytes than that graphene.

On the other hand, the graphyne pores would make it an ideal candidate material for water desalination^31^. Graphyne has plentiful positively charged sites, increasing the oxygen reduction reaction and improving fuel cells' efficiency^32^. Among four types of graphynes, γ‐graphyne has been successfully synthesized, which has high stability, semiconductor characteristic, and widespread application in catalysis and energy‐related fields^29,33^. The theoretical analysis of γ-graphyne has been expanding in recent years. For example, Srinivasu et al.^19^ determined the electronic structure and bandgap energy of γ-graphyne by density functional theory (DFT). Liang et al.^34^ and Kang et al.^35^ investigated the influence of the single-atom defect on γ graphyne by DFT. They noticed an incredible distinction in the electronic and magnetic properties of two forms of γ-graphyne. Yang et al.^36^ developed a one-pot green method for fabricating nitrogen-doped γ-graphyne (NGY) through the ball-milling and calcination processes using NH_4_HCO_3_ as a nitrogen resource. Recently, Henriquede Araujo Chagas et al. used the all-atom molecular dynamics simulations to evaluate the properties of an aqueous mixture of two ionic liquids as an electrolyte for γ-graphyne supercapacitor. They showed that γ-graphyne can be an applicable electrode for use in supercapacitors^37^.

Furthermore, Chen et al.^18^ studied N-, P- and O- doped γ-graphyne as the electrode in supercapacitors. To utilize γ-graphyne in electronics and as an electrode of supercapacitors, tailoring the electronic structure of γ-graphyne by doping or creating defects is critical. In this work, we investigate the γ‐graphyne and its derivatives characteristics as electrode materials for supercapacitors by DFT method. We show how the doped atom (p-type and n-type), its position (sp or sp^2^), and different types of defects directly affect the properties and capacitance of structures. To this end, doped and defective structures' electronic and structural properties, quantum capacitance, and net charge are investigated. The density of states (DOS) and projected/partial density of states (PDOS) plots use to illustrate the electronic properties. The integrated specific quantum capacitance applies to the evaluation of structures' capacitance.

## Computational methods

**Figure 1:**
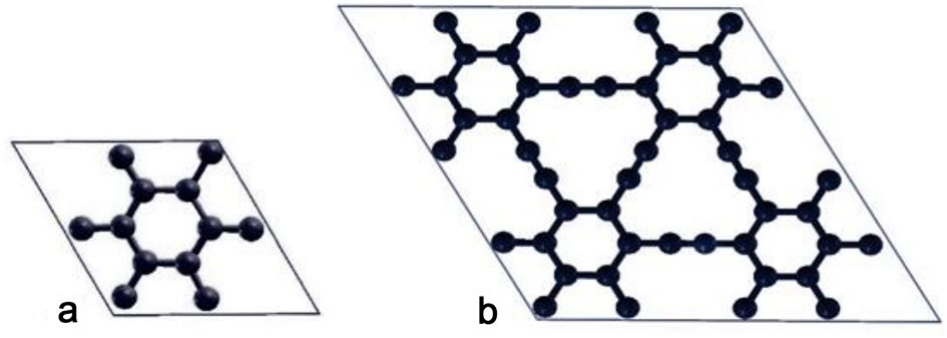
(**a**) unit cell of pristine graphyne, (**b**) 2 × 2 supercell of pristine graphyne.

Initially, we selected a unit cell comprising 12 carbon atoms, as depicted in Fig. 1a. To investigate the impact of doping or defects on the quantum capacitance and cohesive energy, we conducted calculations using 2 × 2 supercells (Fig. 1b). The lattice parameter of pristine graphyne unit cell is optimized at 6.885 Å (or 13.769 Å for its supercell), which matches findings from other studies^38–40^. To examine the electronic properties and optimized geometrical structures of pristine and doped-graphyne sheets, we employed the density functional theory (DFT) framework with the plane wave basis sets and self-consistent field theory (PWSCF)^41^. The calculations were carried out in Quantum Espresso (QE) package^42^. The generalized gradient approximation (GGA) functional type developed by Perdew–Burke–Ernzerhof (PBE)^43,44^ approximates the electronic exchange–correlation energy. We employed the projector augmented wave (PAW)^45^ method to describe the interaction between core and valence electrons. Kohn–sham wave functions have been restricted with an energy cutoff of 100 Rydberg. The Brillouin zone was integrated using a 6 × 6 × 1 Monkhorst– Pack (M–P)^46^ of k-point mesh for geometry optimization and a 12 × 12 × 1 M–P of k-point mesh to obtain the density of state (DOS). The cohesive energy (${E}_{coh}$) was calculated using Eq. (1) to compare the studied structures' relative stability^2,38^. The cohesive energy refers to the required energy to separate the constituent atoms and bring them to an assembly of neutral-free atoms. The more stable structure demonstrates the more negative value of cohesive energy.

1 $${E}_{coh}=\frac{\left({E}_{complex}-(m{E}_{x}+n{E}_{y}+\dots )\right)}{\left(m+n+\dots \right)}$$

where ${E}_{coh}$ refer to cohesive energy, ${E}_{complex}$ is the energy per unit cell of each structure, ${E}_{x}$ and ${E}_{y}$ are the energy of single constituent atoms (the carbon and dopant atoms), and n and m correspond to the number of atoms per unit cell.

Quantum capacitance is significant for non-metal structures, such as some carbon-based electrodes. This parameter refers to the capacitance of an electrode with quantized electronic levels. In principle, quantum capacitance indicates the simplicity of filling quantized levels with charge carriers (i.e., electrons or holes). For two-dimensional crystals, it is defined as follows^5,47^

2 $${C}_{Q }=\frac{d\sigma }{{ d\varphi }_{{\varvec{g}}}}$$

where $d\sigma$ is the change of charge density and ${d\varphi }_{{\varvec{g}}}$ is the changes of graphyne surface potential. Assuming that the Fermi surface is displaced by applying potential and DOS is not affected by the electrode charge, and electrochemical potential $(\mu )$ is rigidly shifted by ${e\varphi }_{{\varvec{g}}}$, the excess charge density $(\sigma )$ can be obtained by^48,49^

3 $$\sigma =e\underset{-\infty }{\overset{+\infty }{\int }}D\left(E\right) \left(f\left(E\right)-f(E-\mu )\right) dE=e{\int }_{-\infty }^{+\infty }D\left(E\right) \left(f\left(E\right)-f(E-{ e\varphi }_{{\varvec{g}}})\right) dE$$

where $D\left(E\right)$ is the density of states, $f\left(E\right)$ is the Fermi–Dirac distribution function, $E$ is the energy concerning *E*_*F*_, $e$ is the elementary charge. Therefore the *C*_*Q*_ is given by^10,27,50^:

4 $${{C}_{Q}}^{diff}=\frac{d\sigma }{{ d\varphi }_{{\varvec{g}}}}={e}^{2} {\int }_{-\infty }^{+\infty }D\left(E\right){F}_{T}\left(E-{ e\varphi }_{{\varvec{g}}}\right)dE$$

where the thermal broadening function ${F}_{T}\left(E\right)$ obtains from the derivative of the Fermi–Dirac distribution function relative to energy and expressed as^27^

5 $${F}_{T}\left(E\right)=-\frac{df}{dE}={\left(4KT\right)}^{-1} {\mathit{sec}h}^{2}\left(\frac{E}{2KT}\right)$$

In this equation, K is the Boltzmann constant, and T is the temperature, considered 300 K. In practical applications, studying the total stored energy is essential for assessing a supercapacitor's efficiency. This quantity is not based on differential capacitance but an integrated and complete charge–discharge cycle. In this way, to anticipate capacitance, we used the integrated quantum capacitance, which can be defined as a function of potential as follows^51,52^

6 $${C}_{Q}^{int}=\frac{Q}{V}=\frac{1}{Ve}{\int }_{0}^{V} {{C}_{Q}}^{diff}\left(V{\prime}\right)d{V}{\prime}$$

## Results and discussion

### Structural and electronic properties of pristine and doped γ-graphyne

**Figure 2:**
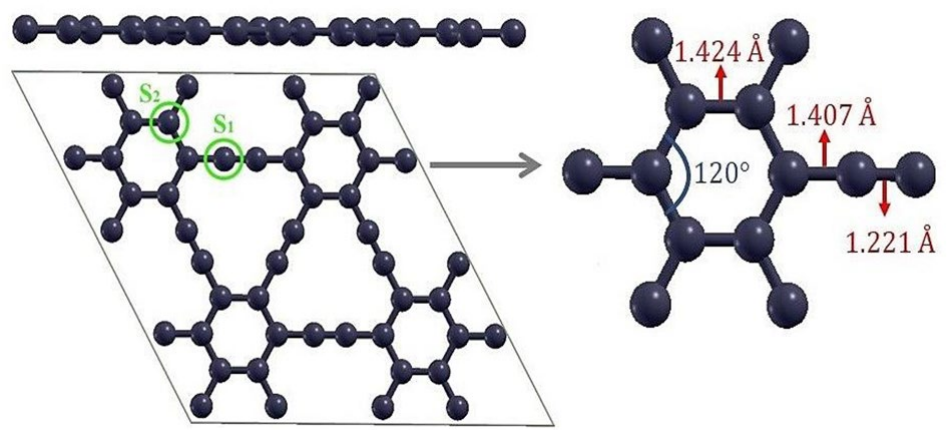
The optimized geometrical structure of pristine *γ-*graphyne. In the left image, S_1_ and S_2_ represent the sp and sp^2^ atoms, respectively. In the right image, the optimized bond lengths and angles are shown.

Figure 2 shows the optimized structure of pure γ-graphyne. After complete relaxation, it is shown that the system maintains its planar form. As claimed, the γ-graphyne network includes two non-equivalent sorts of carbon atoms. The sp^2^-hybridized atoms form the hexagonal rings, and the sp-hybridized atoms form the acetylenic chains and create the pseudo-triangular pore. Three types of bonds can be considered for this structure: (1) triple acetylenic bonds, (2) aromatic double bonds, and (3) the connections between the benzene ring and the acetylene groups (a single bond).

**Figure 3:**
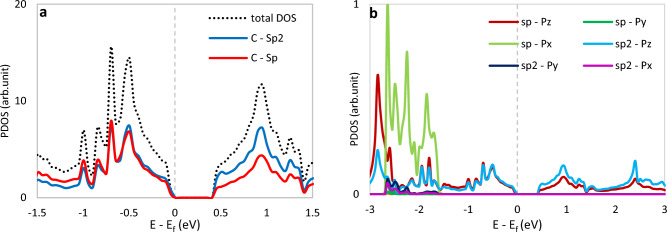
Calculated (**a**) projected density of states and (**b**) partial density of states of pristine γ-graphyne. The Fermi energy has been rescaled at zero.

The results demonstrated that the triple, aromatic, and single bond lengths are 1.221 Å, 1.424 Å, and 1.4070 Å, respectively, which concur with the previous computations^53,54^. Furthermore, generally, the length of a single σ bond, double (σ + π), and triple (σ + 2π) bonds are ∼1.21 Å, ∼1.38 Å, and ∼1.47 Å, respectively. Figure 3 indicates the projected density of states (PDOS) and partial density of states (PDOS) plots of pristine γ-graphyne. As shown, the density of states at the Fermi level is slight, and states near the Fermi level are mainly due to carbon atoms' p orbitals. The contribution of s orbitals is negligible compared to p orbitals and is not shown here.

Furthermore, the results reveal that the contribution of sp^2^ carbon atoms in the conduction band is slightly more significant than sp atoms (see Fig. 3a). Therefore, the electronic character of the structure is more affected by sp^2^ carbons. All the more explicitly, the states near the Fermi level are mainly contributed by the carbon's p_z_ orbitals. In contrast, the p_x_ and p_y_ orbitals begin adding states in the valence and conduction bands far from the Fermi energy.

**Figure 4:**
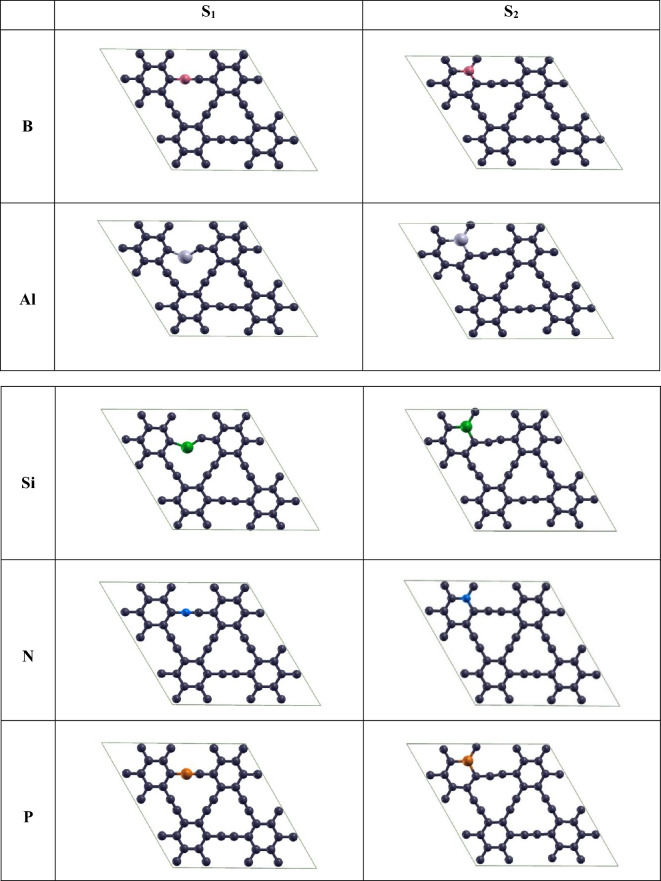
Top view of optimized structures corresponding to substitutional doping with B, Al, Si, N, and P in S_1_ (sp site) and S_2_ (sp^2^ site) of pristine graphyne (The black, pink, gray, green, blue, and orange atoms represent C, B, Al, Si, N, and P atoms, respectively).

The lack of DOS at the Fermi level demonstrates semiconductivity of γ-graphyne. Numerous theoretical and experimental studies show that doping with atoms can change carbon nanomaterials' properties. Depending on the coexistence of two types of C atoms in graphyne structure, we can consider two distinct situations for doping a heteroatom, signified by S_1_(sp) and S_2_ (sp^2^), which appear in Fig. 2. We have investigated the change of electronic properties and quantum capacitance induced by doping with Al, B, Si, N, and P in these two circumstances, as shown in Fig. 4.

Among these five atoms, B and Al are p-type dopants. The p-type dopant has one electron less than carbon, leading to the electron-free cavity in the valance band of the closed-shell structure. Since this impurity is willing to accept an electron, it is known as an acceptor. On the other hand, N and P atoms are n-type dopants. The four outer electrons link with the carbon atom, while the fifth electron is in free movement and acts as a charge carrier. This free electron requires considerably less energy to move from the valence band to the conduction band. These impurities that enhance the carrier density by contributing extra electrons to the conduction band are called donors. The silicon atom is in the same group as the carbon atom and only has a larger atomic radius relative to carbon (Si 1.11 Å and C 0.77 Å). The bond lengths, cohesive energies, and electronic properties of S_1_ and S_2_- doped graphyne (after the optimization) are listed in Tables 1 and 2, respectively.

**Table 1:** Electronic character, optimized bond lengths, and cohesive energy of pristine and S_1_-doped graphyne.

	Dopant	Cohesive energy(eV)	Bond hybrid	Bond length (Å)	Electronic character
*Pristine* *γ-Graphyne*	–	− 8.564	C_sp_-C_sp_	1.2214	Semiconductor
			C_sp2_-C_sp_	1.4070	
			C_sp2-_C_sp2_	1.4245	
*sp-doped* *γ-Graphyne*	**B**	− 8.489	B-C_sp_	1.3407	Metal
			C_sp2_-B	1.4870	
			C_sp_-C_sp2_	1.3596	
			C_sp2_-C_sp2_	1.4248	
	**Al**	− 8.383	Al-C_sp_	1.8447	Metal
			C_sp2_-Al	1.9201	
			C_sp_-C_sp2_	1.3651	
			C_sp2_-C_sp2_	1.4072	
	**Si**	− 8.485	Si-C_sp_	1.6617	Semiconductor
			C_sp2_-Si	1.8023	
			C_sp_-C_sp2_	1.3746	
			C_sp2_-C_sp2_	1.4115	
	**N**	− 8.550	N-C_sp_	1.1826	Metal
			C_sp2_-N	1.3584	
			C_sp_-C_sp2_	1.3872	
			C_sp2_-C_sp2_	1.4300	
	**P**	− 8.459	P-C_sp_	1.6179	Metal
			C_sp2_-P	1.8441	
			C_sp_-C_sp2_	1.3568	
			C_sp2_-C_sp2_	1.4129

**Table 2:** Electronic character, optimized bond lengths, and cohesive energy of pristine and S_2_-doped graphyne.

	Dopant	Cohesive energy (eV)	Bond hybrid	Bond length (Å)	Electronic character
*Pristine* *γ-graphyne*	–	− 8.564	C_sp_-C_sp_	1.2214	Semiconductor
			C_sp2_-C_sp_	1.4070	
			C_sp2-_C_sp2_	1.4245	
*sp*^*2*^*-doped* *γ-Graphyne*	**B**	− 8.510	C_sp_-C_sp_	1.2275	Metal
			C_sp2_-C_sp_	1.3898	
			C_sp2_-B	1.5296	
			B-C_sp_	1.4925	
	**Al**	− 8.393	C_sp_-C_sp_	1.2366	Metal
			C_sp2_-C_sp_	1.3672	
			C_sp2_-Al	1.8685	
			Al-C_sp_	1.8057	
	**Si**	− 8.449	C_sp_-C_sp_	1.2252	Semiconductor
			C_sp2_-C_sp_	1.3863	
			C_sp2_-Si	1.7567	
			Si-C_sp_	1.7142	
	**N**	− 8.527	C_sp_-C_sp_	1.2275	Metal
			C_sp2_-C_sp_	1.3895	
			C_sp2_-N	1.4174	
			N-C_sp_	1.3482	
	**P**	− 8.347	C_sp_-C_sp_	1.2244	Metal
			C_sp2_-C_sp_	1.3833	
			C_sp2_-P	1.7112	
			P-C_sp_	1.6319	

The results revealed that the structure retained its planar shape after B-doping in both S_1_ and S_2_ cases. Nevertheless, the bond lengths increased at the doping position compared to the pristine graphyne. It appears that the C-B bond length has expanded about 0.119 Å and 0.075 Å compared to the C–C, respectively, for sp and sp^2^. For the S_2_ one, the angle between the atoms in the benzene ring at the doped site decreases from 120° to 117.590° and between the adjacent atoms to 119.171°. Subsequently, the entry of boron dopant extends the lattice and lessens in-plane stiffness. As a result, the cohesive energy for the B-doped graphyne (BGY) in the S_2_ site is 0.021 eV, more negative than the other in the S_1,_ which indicates a little more stability of S_2_ than S_1_.

Because of its larger atomic radius, aluminum influences the structure geometry and causes bond length increments at the doped site. When Al substitutes the C atom in γ-graphyne, it brings local strain around the doping site and folds the Al-doped γ-graphyne (AlGY) plane. As a result, the bond length at the S_1_ site increases from 1.221 Å in pristine graphyne ($\mathrm{C}\equiv \mathrm{C}$) to 1.845 Å in Al-doped structure ($\mathrm{Al}\equiv \mathrm{C}$), and the bond of the aluminum-benzene ring increases from 1.407 Å to 1.920 Å. In S_2_, bond lengths into the aromatic rings increase from 1.424 Å in graphyne to 1.868 Å in the Al-doped structure, but no protuberance was seen from the surface. In the quasi-triangular cavity, the triple bond length is slightly increased (up to 0.01 Å), which causes slight changes in the geometric structure of this area. According to the results, for the case of aluminum-doped graphyne in the S_1_ site, we observed a 51.17% increase in Al≡C bond length compared to the same position in pure graphyne, which makes the Al atom be over the surface. For the S_2,_ a 31.03% increment was observed in the Al=C bond length. To compare the stability of structures, the cohesive energy was obtained for S_1_-AlGY, − 8.383 eV, and S_2_-AlGY, − 8.393 eV. Thus, the stability of the S_1_-AlGY is slightly less than that of the S_2_-AlGY, and both are less than that of pristine graphyne. When the C atom supplants with Si, the graphyne's geometrical structure is transformed, especially in the S_1_ case because of the larger radius of Si. Replacing an sp carbon atom (S_1_) with a Si atom changes the triple Si≡C bond length from 1.221 Å to 1.661 Å and changes the angle of acetylenic atoms from 180° to 126.876°. This change in bond length and angle has been attributed to the hybridization change of the Si atom from sp to sp^3^^55^.

Entering this dopant atom reduces the length of the C–C bond attached to the sp carbons (1.375 Å) and increases the bond of Si-sp^2^ carbon (1.802 Å). In the S_2_ case, the length of the carbon-silicon bond (compared to 1.424 Å, the length of the aromatic carbon–carbon bond in pristine graphyne) changes to 1.756 Å. Likewise, the angle inside the ring decreases from 120° to 113.989° in the doping position. Cohesive energy for S_1_-SiGY (silicon-doped graphyne), − 8.485 eV, and S_2_-SiGY, − 8.449 eV, was obtained, reflecting the same stability of S_1_ and S_2_ states.

Nitrogen has the closest atomic radius to carbon and is suitable for doping in carbonic structures. By N-doping, the change of bond lengths and angles, particularly in the S_2_ site, is negligible, and a slight change is observed in the geometric structure. When the nitrogen atom is doped in the S_1_ site, the length of the nitrogen-carbon bond at the triple bond changes from 1.221 Å to 1.358 Å, and the nitrogen bond with the benzene ring decreases to 1.183 Å. At the S_2_ site, nitrogen entering causes the bond length to be slightly reduced from 1.424 Å in pristine graphyne to 1.417 Å in a doped structure. The cohesive energy of N-doped graphyne (NGY) was acquired at − 8.55 eV for the S_1_ site and − 8.53 eV for the S_2_ site. It indicates that substitution of nitrogen atom instead of an sp carbon atom gave the structure a little more stability than replacement with sp^2^ carbon. The presence of nitrogen impurities causes the carbon lattice to shrink and increment the in-plane stiffness.

The phosphorus atom has a larger atomic radius than carbon and nitrogen atoms. So, graphyne geometry are affected by doping with P atom. The cohesive energy for P-doped graphyne (PGY) at the S_1_ site was obtained, − 8.459 eV, and at the S_2_, − 8.347 eV, so S_1_-PGY is more stable than S_2_-PGY. S_2_ doped state preserves its planar geometry, but doping in the S_1_ position destroys the planar structure. Replacement of the phosphorus atom instead of carbon in the benzene ring increases the length of the P–C bond to 1.711 Å and decreases the angle between the phosphorus and the two side carbons from 120° to 114.928°. At the S_1_ site, the bond length of the phosphorus and adjacent sp carbon atom changes from 1.221 Å to 1.618 Å and the connection between phosphorus and benzene ring changes from 1.407 Å to 1.357 Å. In this case, the phosphorus atom has the highest height among all atoms in the cell and is located at 1.376 Å from the structure's surface.

**Figure 5:**
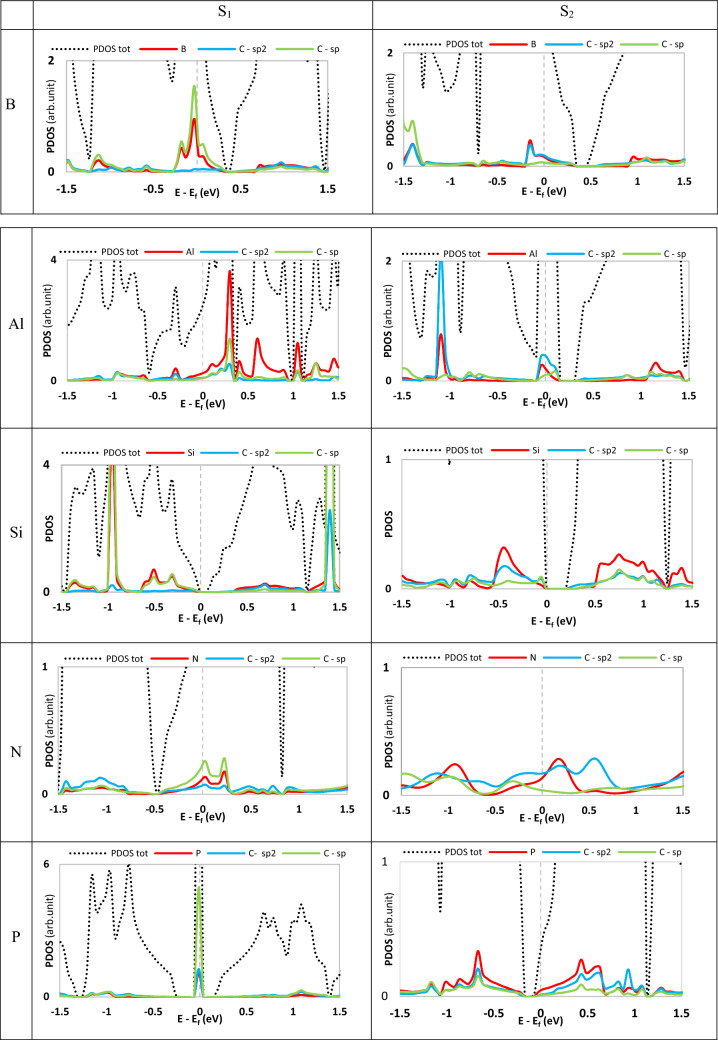
Projected density of states of doped graphyne. S_1_ and S_2_ panels are sp and sp^2^ atoms doping positions, respectively. The Fermi energy has been rescaled at zero. In the state of Si-doped in the S_2_ position, the total PDOS is in the out-of-defined axes range. So, it is not seen in the plot.

Figure 5 shows the PDOS plots of the mentioned structures. As shown, all the structures got metallic except the silicon-containing system. In B-doped graphyne, the sp carbon atom adjacent to the boron atom in the S_1_ site, and the boron atom itself plays a significant role in producing new states. Furthermore, the contribution of the adjacent sp^2^ carbon atom at the illustrated interval is lower, and the effect of farther atoms is negligible. In the S_2_ case, the boron atom and the neighboring sp^2^ carbon contribute approximately equally to forming the states near the Fermi level. The PDOS plot of aluminum-doped graphynes shows that the doping with an aluminum atom in the S_1_ site makes a sharp peak at about 0.3 eV. Herein, unlike B-doping, the Al atom leads to creating the new states in the Fermi level for both adjacent sp and sp^2^ carbon atoms. In the S_2_-AlGY structure, the contribution of one adjacent sp^2^ carbon atom in the specified energy range is also higher than the Al atom, and Al is just slightly more effective at about 1.154 eV. For Si-doped graphyne, we found that the silicon atom creates electronic states similar to the adjacent sp carbon when placed in the S_1_ position. In contrast, the Si atom has a more influential performance than other atoms in the S_2_ site.

Nevertheless, it can be seen that Si does not change the electronic character of the structure because of a closed-shell electron configuration. For P-doped graphyne, in the S_1_ position, the sharp peak of states comes from the sp carbon atoms adjacent to the phosphorus, and adjacent sp^2^ carbon atoms have a much lower contribution in forming new states. In the S_2_ position, the phosphorus atom creates more new states at and around the Fermi surface than neighboring carbon atoms.

### Quantum capacitance of pristine and doped γ-graphyne

**Figure 6:**
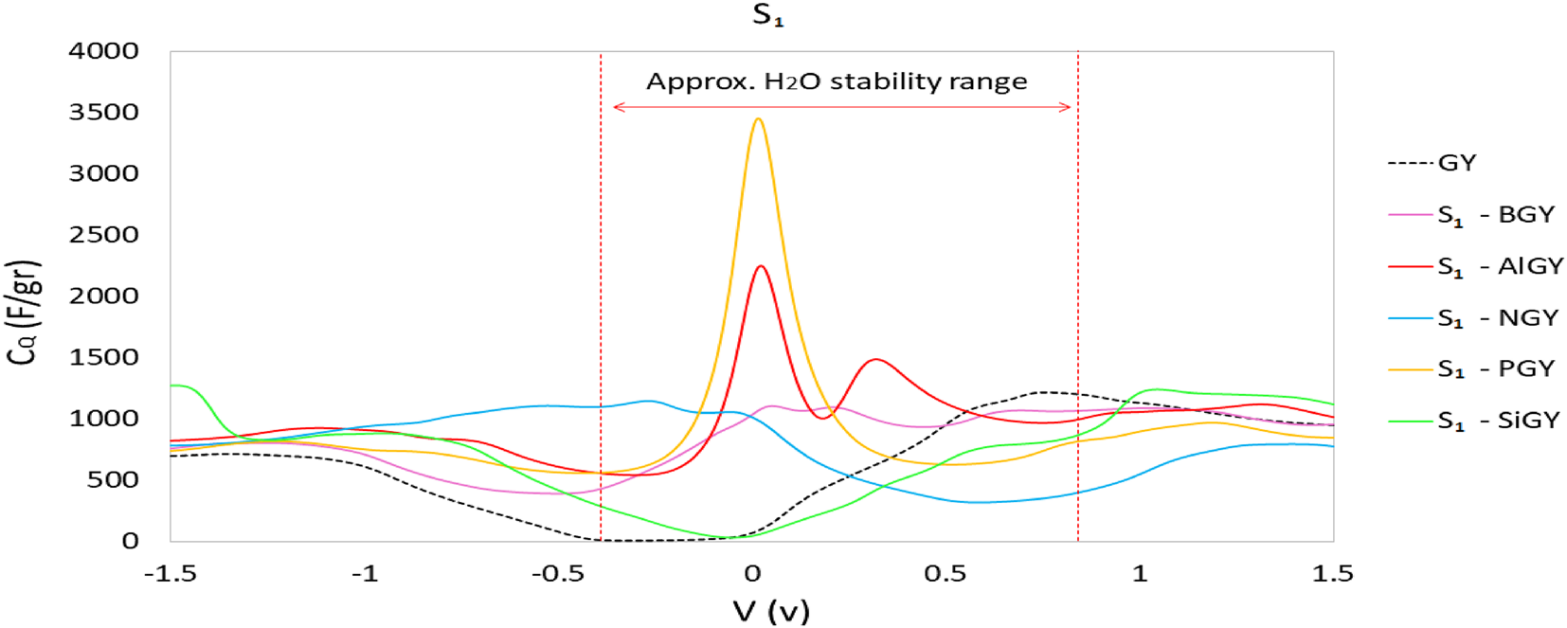
Integrated specific quantum capacitance of undoped and S_1_-doped graphyne. S_1_ is the position of the sp atom.

**Figure 7:**
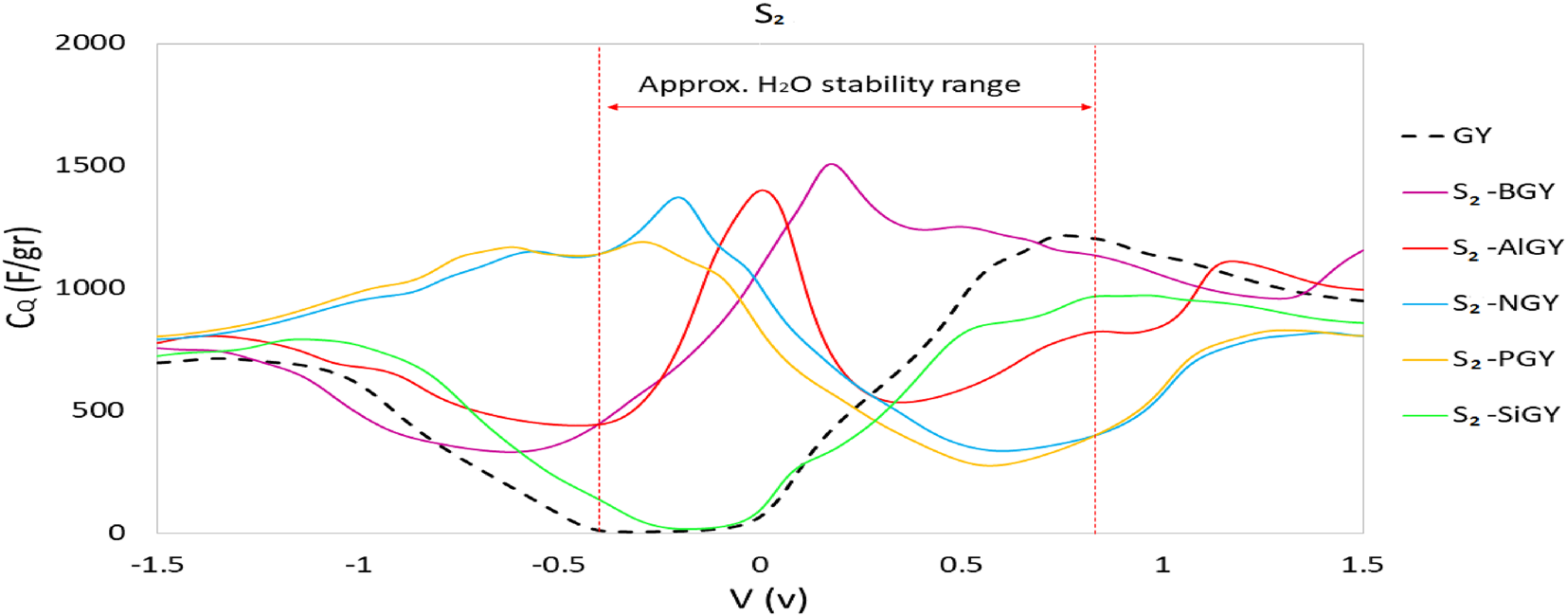
Integrated specific quantum capacitance of undoped and S_2_-doped graphyne. S_2_ is the position of the sp^2^ atom.

Given what has been said, since quantum capacitance is straightforwardly related to the electronic density of states (Eq. 3), we anticipate that the amount of ${C}_{Q}$ should increment after doping. So, we have determined the specific integrated quantum capacitances (signified by ${C}_{Q}$) of pristine and doped γ-graphyne appear for S_1_-doped graphyne (Fig. 6) and S_2_-doped graphyne (Fig. 7). According to the integrated quantum capacitance diagram in these figures, except Si, all doped atoms improved ${C}_{Q}$ around zero potential.

The results reveal that boron atoms have improved the capacitance in both S_1_ and S_2_ positions. The capacitance difference is slight at the beginning and end of the approximate stability potential of water. Nevertheless, a better quantum capacitance has been obtained by substituting the boron atom in the S_2_ site relative to the S_1_ site The maximum quantum capacitance observed for the S_1_ case is 1105.82 F/gr at 0.052 V, and the S_2_ is 1508.65 F/gr at 0.177 V. Since the maximum quantum capacitances are seen for both cases at positive bias, BGY can be a good choice for a positive electrode in asymmetric supercapacitors. Doping with aluminum improves the quantum capacitance at zero potential significantly. The maximum S_1_-AlGY quantum capacitance is 2251.10 F/gr at 0.022 V, and for S_2_-AlGY is 1401/64 F/gr at 0.005 V, which is positive and very close to zero for both structures. As indicated in Figs. 6 and 7, the Al-doped structure has a more symmetrical diagram, which can be said that this structure can function well as a symmetric supercapacitor electrode. SiGY has ${C}_{Q }\approx$ 0 at negative bias near the zero potential, similar to graphyne. Although the substitution of the Si atom has not improved quantum capacitance around zero potential and the maximum ${C}_{Q}$ for both cases (S_1_ and S_2_) is less than pristine graphyne. A slight improvement in the negative potentials can be seen, which is less than required.

The maximum ${C}_{Q}$ of NGY, in the approximate range of water stability potential, was obtained at 1146.48 F/gr at − 0.27 V for the S_1_ position and 1372.84 F/gr at − 0.20 V for the S_2_ position. In addition to that, the ${C}_{Q}$ of NGY in the two different sites at different potentials were nearly identical, and both performed better at negative potentials. NGY is proposed as a negative electrode in asymmetric supercapacitors based on these results.

Figure 6 shows that S_1_-PGY has a symmetrical quantum capacitance with a maximum value of 3454.16 F/gr at 0.02 V. However, this high capacitance value drops sharply in positive and negative potentials, and this structure only in a small range of potential shows high quantum capacitance. As a result, the ${C}_{Q}$ at the beginning and end of the water stability potential range is significantly lower. S_2_-PGY also has a maximum quantum capacitance of 1192.77 F/gr at − 0.29 V.

**Figure 8:**
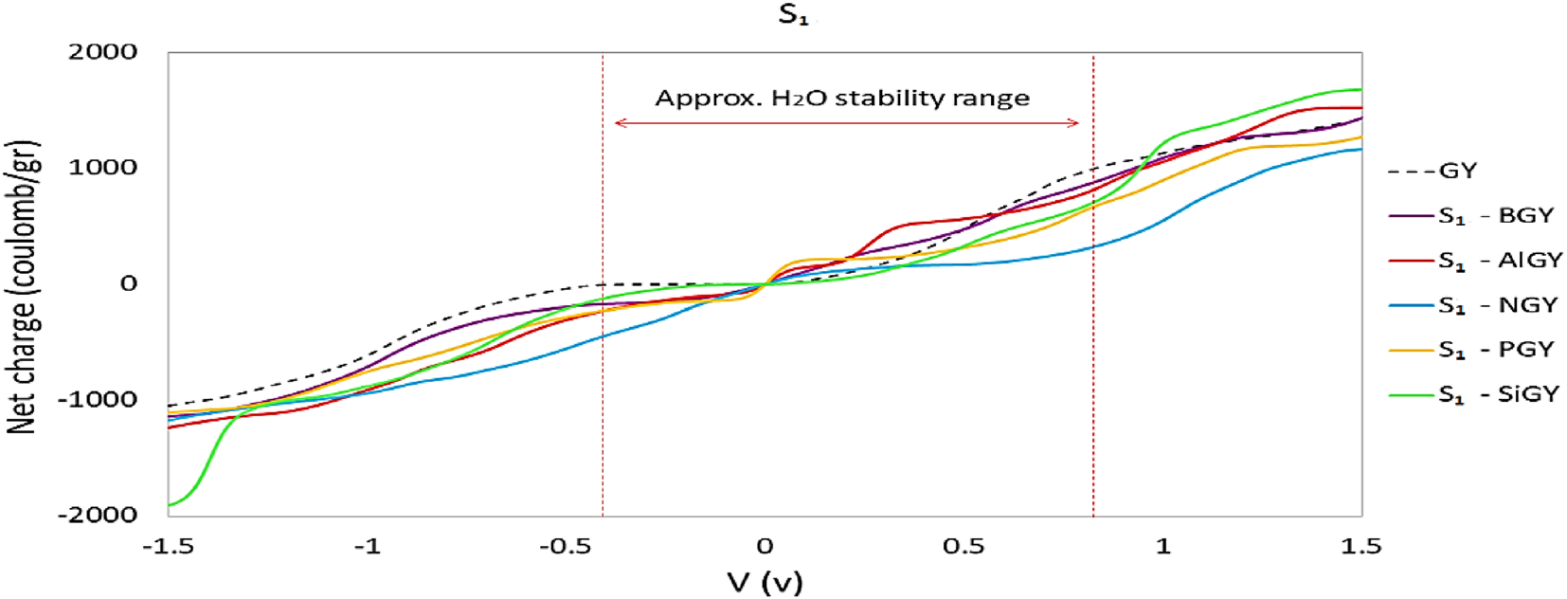
Calculated net charge on undoped and S_1_- doped graphyne.

**Figure 9:**
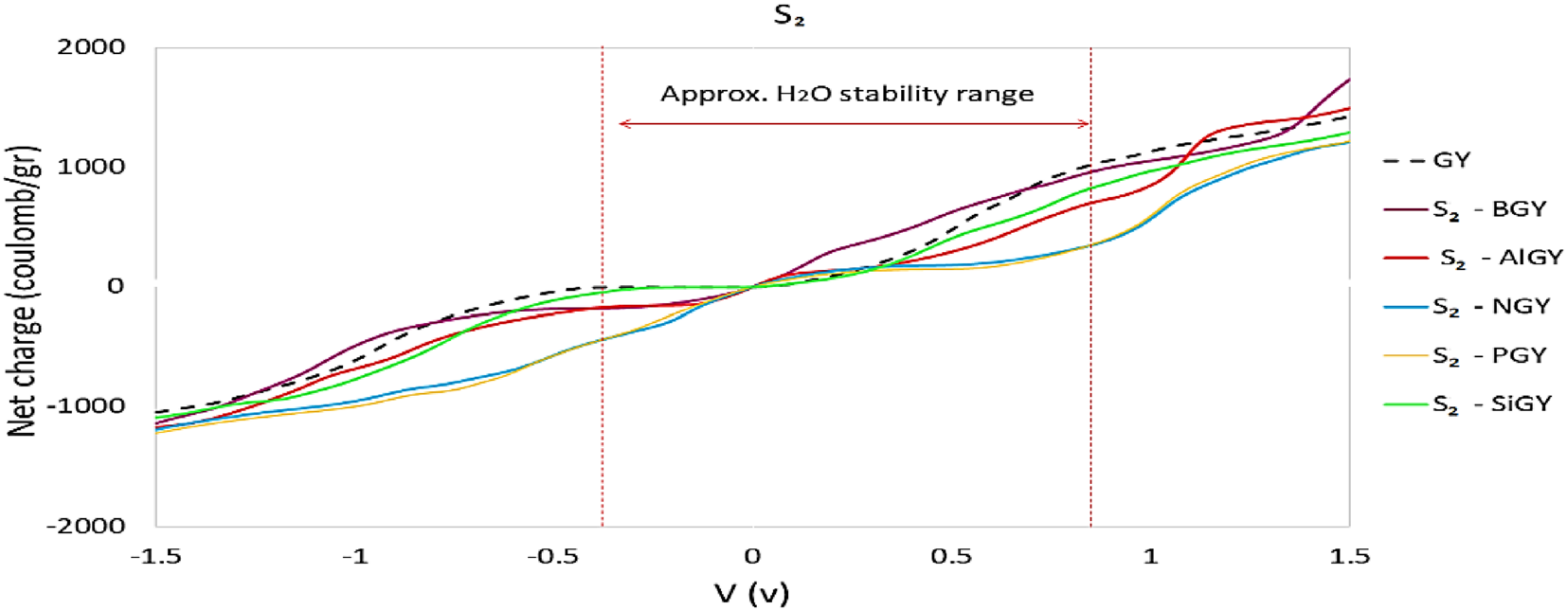
Calculated net charge on undoped and S_2_-doped graphyne.

Figures 8 and 9 represent the calculated net charge on doped graphyne systems as supercapacitor electrodes. According to Fig. 8, all doped atoms except Si improved the quantum capacitance near zero potential. Among the S_1_ cases, nitrogen-doped as the cathode, and boron and aluminum-doped can be used as anode in asymmetric supercapacitors. Phosphorus-doped graphyne in the S_1_ site with symmetrical behavior is suitable for symmetric supercapacitors electrode. According to Fig. 9, in the S_2_ position, nitrogen and phosphorus-doped graphyne perform better at negative potentials and are recommended as cathodes in asymmetric supercapacitors. In contrast, S_2_-BGY performs better at positive bias and is more suited as an anode. In symmetric supercapacitors, S_2_-AlGY can also be used as an electrode.

### Structural and electronic properties of defective γ-graphynes

**Figure 10:**
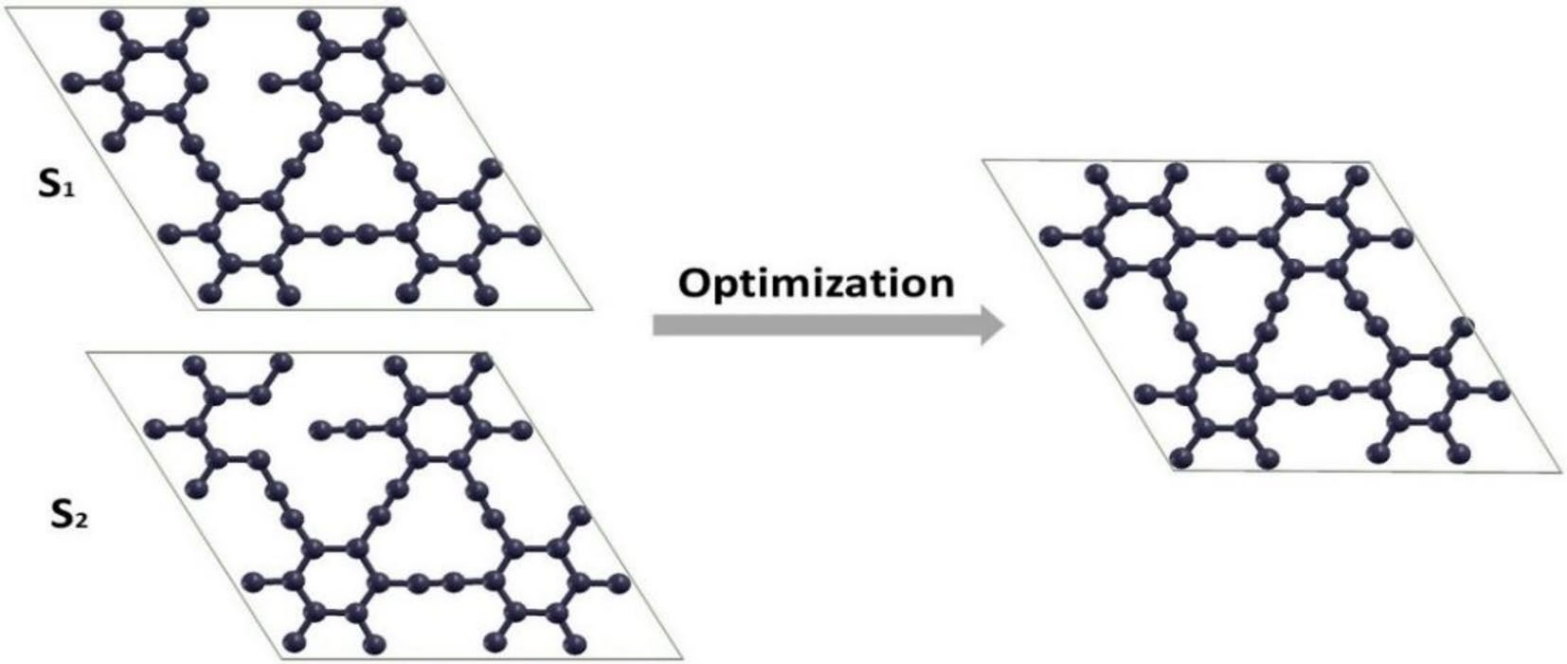
Top view of structures corresponds to V_1_-sp and V_1_-sp^2^ before and after optimization processes.

The creation of defects is inescapable during material production. These defects significantly affect structures' electronic, optical, thermal, and mechanical properties (graphyne here). Therefore, we have considered removing one and two carbon atoms from both S_1_ and S_2_ sites to create defective graphyne. Our findings demonstrated that an in-plane rearrangement occurs after optimizing the defective graphyne by removing a sp or sp^2^ carbon atom (see Fig. 10). As shown, by removing one sp atom, the neighboring carbons stretch in the defect position and form a new bond with the sp^2^ carbon atom of the ring. Also, when a sp^2^ carbon atom is removed, its adjacent atoms move to the vacant position and form a new covalent bond. Therefore, the acetylenic bond, with a bond length of 1.222 Å, stretches and increases to 1.427 Å in the defective structure near the defect site after optimization steps. The aromatic bonds of the two rings adjacent to the defect region also changed from 1.424 Å to 1.484 Å. However, there were no bumps or indentations on the structure's surface, and the system stayed planar. The defect in the S_2_ position causes identical changes in bond lengths and angles similar to the optimized structure of the defective graphyne in the S_1_ site. Both systems had the same cohesive energy and stability, equaling − 8.478 eV, indicating that none is superior. However, after creating defects and optimization, the symmetry of graphyne has altered, diminishing its stability compared to the pure structure Fig. 9 shows the V_1_-sp and V_1_-sp^2^ structures before and after the optimization processes. The point defect is represented with V_1_.

**Figure 11:**
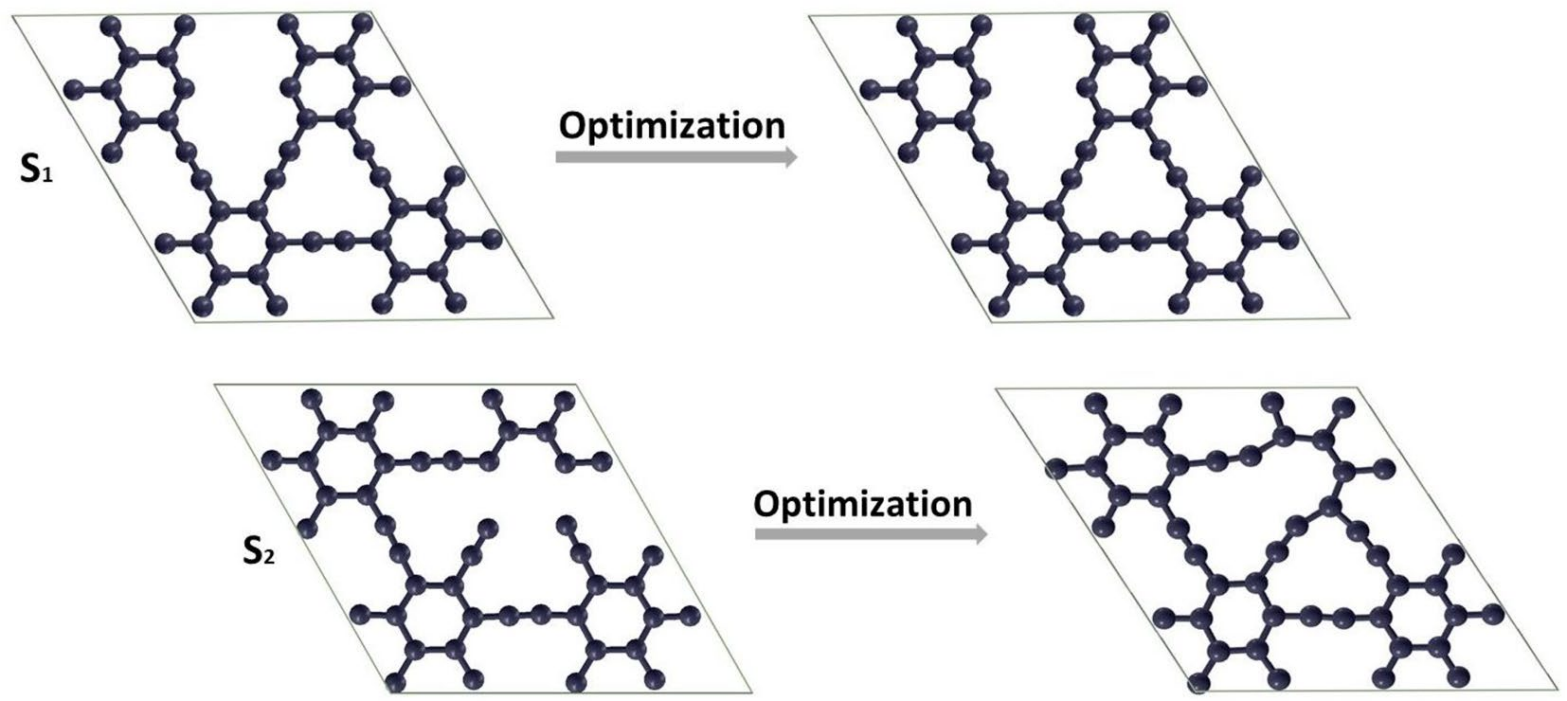
The top view of structures corresponds to V_2_-sp (removing two sp carbon atoms) and V_2_-sp^2^ (two sp^2^ carbon atoms) before and after optimization processes.

Following these two cases, the defect was examined by removing two carbon atoms (see Fig. 11, S_1_), which are indicated in the plots with V_2_. For this case, no in-plane rearrangement was seen after optimizing for the S_1_-defective structure. The bond length in the aromatic ring in the two benzene rings closest to the defect area was altered (from 1.424 Å to 1.385 Å). The changes in bond lengths of triple bonds around the defect position were negligible. The calculated cohesive energy of the optimized planar structure was − 8.457 eV, demonstrating that it is less stable than pristine graphyne.

In the other case, in the absence of two carbon atoms at the S_2_ position, the sp carbon atoms adjacent to the defect site approach the near sp^2^ atoms and form new bonds (see Fig. 11, S_2_). As indicated by the outcomes, the bond lengths of the majority of atoms change after optimization. Despite these changes, the structure retains its planar geometry. The cohesive energy of this structure was achieved at − 8.464 eV, which shows that this type of defect is somewhat more stable than the S_1_ one. The new bonds have a 1.454 Å bond length, were created after optimization, and the angle between sp^2^ atoms in opened benzene 142.121° was obtained. Acetylenic bonds near the defect site, which were 1.221 Å before making the defect, also changed to 1.248 Å. The angle between them also decreased from 180° to 157.104°. The optimized structure for both V_2_ defects is shown in Fig. 10.

**Figure 12:**
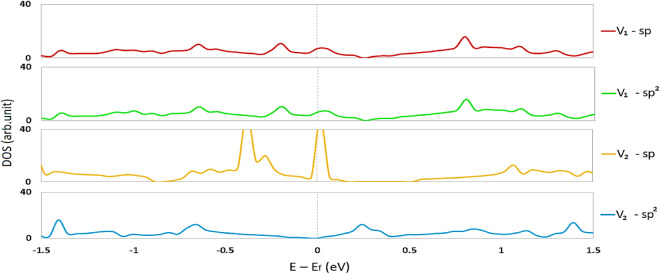
The density of states of V_2_-sp (removing two sp carbon atoms) and V_2_-sp^2^ (two sp^2^ carbon atoms) defective graphynes. The Fermi energy has been rescaled at zero.

For V_1_ defects, as represented in Fig. 12, removing each of the sp or sp^2^ atoms from the graphyne structure can create new states at the Fermi level. Since the optimized structure for both single-atom vacancy graphynes is the same, the density of states diagram also shows the same electron properties for both structures, so we anticipate that they should have a similar quantum capacitance (V_1_-sp and V_1_-sp^2^ in Fig. 12). Due to removing two sp carbon atoms (V_2_-sp in Fig. 12), two significant peaks appear, one at the Fermi level and the other around − 0.5 eV. Nevertheless, no peaks are observed by separating the two sp^2^ carbon atoms from the graphyne structure at the Fermi level (V_2_-sp^2^ in Fig. 12). We expect a quantum capacitance diagram similar to the pure graphyne.

### Quantum capacitance of defective γ-graphynes

**Figure 13:**
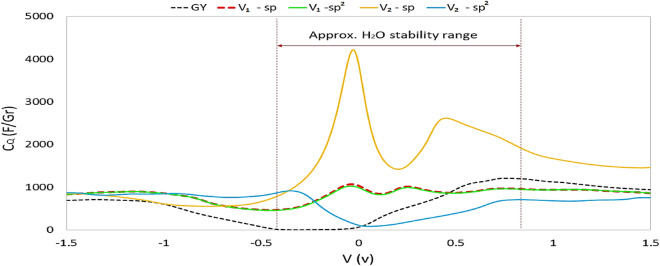
Quantum capacitance of different types of defective graphynes.

According to Fig. 13, as we guessed from the DOS diagram, removing one carbon atom (point defect) from the structure (sp or sp^2^) gives almost the same quantum capacitance result regardless of the initial defect location. In this case, the capacitance behavior is relatively favorable, and in the stability range of water, the structure has maintained its capacitance. The maximum quantum capacitance for the V_1_-spGY was equal to 1037.23 F/gr at − 0.04 V, and the sp^2^ one was equal to 1030.44 F/gr at − 0.44 V. This difference is probably related to the calculation errors. This difference is probably related to the calculation errors. Removing two sp carbon atoms improved the quantum capacitance at zero and positive potentials. The maximum quantum capacitance for this structure is 4221.69 F/gr, which is observed at − 0.03 V. Removing two sp^2^ carbon atoms leads to a defect that does not exhibit favorable quantum capacitance and thus may not be appropriate for use in water-electrolyte supercapacitors. So, as per Fig. 13, the V_2_-sp structure can be a suitable material as an anode electrode in asymmetric supercapacitors. While The structures with a single atom defect (V_1_) in both cases are suggested for the symmetric supercapacitor electrode.

## Conclusion

We have investigated the doping and defect effects on γ-graphyne by density functional theory calculations. The results reflect that substituting all p-type and n-type dopants (B, Al, N, and P) instead of one of sp or sp^2^ carbon atoms in γ-graphyne positively affects the stability, electronic properties, and quantum capacitance. In this manner, such changes can obtain various quantum capacitance and properties from graphyne. All investigated dopants except Si improved quantum capacitance near zero potential. S_1_- and S_2_-NGY are recommended as the cathode, and S_1_- and S_2_- BGY as the anode among studied structures. Nevertheless, S_2_-PGY shows a symmetrical behavior. So, it is suitable for symmetric supercapacitors. In contrast, S_1_-PGY is recommended as the cathode in asymmetric supercapacitors. In contrast, aluminum in the S_1_ position makes the structure more suitable for the anode, but S_2_-AlGY can perform as an electrode in symmetric supercapacitors. Despite less stability related to other defective cases, evaluations of defective cases have shown that the V_2_-sp structure seems desirable regarding electronic properties and quantum capacitance. However, the structure of V_2_-sp^2^ does not have favorable electronic properties as a supercapacitor electrode. Creating a point defect in the structure of graphyne can likewise give good outcomes for its utilization as supercapacitor electrodes. Finally, the maximum quantum capacitances obtained for the doped and the defected structures are 2251.10 F/gr and 4221.69 F/gr (related to AlGY doped in the sp position and structure with removing two sp carbon atoms, respectively), which are the values more than most of the structures proposed so far^4,10,18,56–58^.

## Data Availability

The datasets generated during the current study are available from the corresponding author on reasonable request.

## Abbreviations

DFT: Density functional theory
2D: Two-dimensional
DOS: Density of states
PDOS: Projected/partial density of states
GY: Graphyne
